# Sleep Modulates Emotional Effect on False Memory

**DOI:** 10.11621/pir.2022.0110

**Published:** 2022-03-30

**Authors:** Ruchen Deng, Aitao Lu

**Affiliations:** a Key Laboratory of Brain, Cognition and Education Sciences, Ministry of Education, China; School of Psychology, Center for Studies of Psychological Application, and Guangdong Key Laboratory of Mental Health and Cognitive Science, South China Normal University, China

**Keywords:** False memory, sleep, emotion, mood, DRM

## Abstract

**Background:**

Whereas sleep and emotion are important factors affecting false memory, there is a lack of empirical research on the interaction effect of sleep and emotion on false memory. Moreover, it should be investigated further that how the effects of emotion on false memory varies from presenting emotional content to eliciting emotional state.

**Objective:**

To examine how sleep and varying emotional context influence false memories. We predicted that sleep and emotion would interactively affect false memory when participants are presented with negative words in a learning session (Experiment 1) or when their emotional state is induced before a learning session (Experiment 2).

**Design:**

We used the Deese-Roediger-McDermott (DRM) task. Emotional words were used to elicit emotion during learning in Experiment 1 and video clips were used to induce a particular mood state before learning in Experiment 2. Participants were divided into a “sleep group” and a “wake group” and completed an initial learning session either in the evening or in the morning respectively. After a learning session, participants in the sleep group slept at night as usual and completed a recognition test in the morning, while participants in the wake group stayed awake during the daytime and completed their recognition test in the evening. All participants completed a recognition test after the same period of time.

**Results:**

In Experiment 1, the wake group falsely recognized more negative critical lure words than neutral ones, but no such difference existed in the sleep group, suggesting that sleep modulated the emotional effect on false memory. In Experiment 2, participants in either a positive or negative mood state showed more false recognition than those in a neutral state. There was no such difference in the wake group. We conclude that sleep and emotion interactively affect false memory.

## Introduction

Human memory is prone to distortions and is easily influenced by a multitude of factors such as sleep (e.g., Diekelmann et al., 2009; Payne et al., 2009) and emotional valence (e.g., Bookbinder & Brainerd, 2017; McKeon et al., 2012). Memory distortion in such cases is often referred to as false memory. Many situations in everyday life can produce false memory. For example, people can falsely recall childhood events, and through effective suggestions, they can even create new false memories. In the laboratory, the Deese-Roediger-McDermott (DRM) paradigm (Deese, 1959; Roediger & McDermott, 1995) has been widely used to induce false memory (e.g., Huan et al., 2021; McKeon et al., 2012; Roediger, Watson et al., 2001). In this paradigm, participants study lists of words (e.g., winter, snow) that are semantically associated with a non-presented critical lure word (e.g., cold). In the test session, participants consistently recall or recognize the non-presented lure with the same level of confidence as the correctly remembered studied words (Roediger & McDermott, 1995; Roediger, Watson et al., 2001).

According to fuzzy-trace theory (FTT; Reyna & Brainerd, 1995), when an event is experienced, two parallel traces are stored: a verbatim trace, which preserves item-specific and contextual information, and a persistent gist trace, which is based on the extraction of the general meaning of the encoded information. High false acceptances of critical lures in the DRM paradigm are explained as resulting from the similarity of gist traces between the list-items and the critical lure (e.g., Brainerd & Reyna, 2002). An alternative theory that has been proposed to explain the DRM illusion is activation/monitoring theory (AMT; Roediger, Balota et al., 2001). This theory poses two complementary processes that lead to false memories: automatic activation of critical lures and a breakdown in source monitoring. A key difference between these two theories is that, in the first case, gist extraction is due to semantic attributes emerging from the list structure. In the latter case, associative activation is caused by the statistical co-occurrence of items in the mental lexicon. In general, these theories make similar predictions for DRM research. However, there is some evidence that semantically related items that have no associative relation (e.g., butter) can induce lure false alarms (e.g., salt; Brainerd et al., 2008), suggesting that the manipulation of semantically related items could reliably and easily produce false memory, though such semantic manipulation would be controversial.

What factors affect the emergence of false memory? The key role of sleep in memory processing has recently drawn the interest of a growing number of researchers (e.g., Calvillo et al., 2016; Chatburn et al., 2017; Lo et al., 2016; Pardilla-Delgado & Payne, 2017). However, results from previous studies in the field are inconclusive. While some studies showed that sleep had an enhanced impact on the generation of gist memories, others found that sleep had no effect on or even reduced the storage of such memories in comparison to wakefulness. For example, Darsaud et al. (2011) found an enhanced recognition of false memories following sleep as compared to sleep deprivation, whereas Diekelmann et al. (2008) reported the opposite: reduced false memories after sleep as compared to sleep deprivation.

Emotion is another important factor assumed to play an active role in memory consolidation. The relationship between emotion and memory has been extensively studied in the context of eyewitness testimony (e.g., Kaplan et al., 2016), and the general conclusion is that emotion can either support or impair memory. On the one hand, previous research found that high-arousal emotional states or learning materials evoked more false memories regardless of valence characteristics (e.g., Corson & Verrier, 2007), mainly because the highly evoked emotional information during coding narrows the attention span, causing people to focus only on the main idea clues and to ignore the peripheral details (e.g., Bookbinder & Brainerd, 2017; Kaplan et al., 2016). On the other hand, some studies found that emotions tend to capture attention, thereby prioritizing processing, leading to better encoding and enhanced memory (e.g., Dolcos & Denkova, 2014; Pourtois et al., 2013).

Such inconsistent results from previous studies on sleep and emotion show that more evidence is needed. Additionally, many prior studies have confirmed that sleep and emotion are strongly associated. With respect to the influence of sleep on emotion, the emotional regulation theories of sleep suggest that sleep is an important mechanism affecting emotional responses to stressors (e.g., Deliens et al., 2014; Goldstein & Walker, 2014). Neuroimaging studies have shown that individuals exhibit an exaggerated amygdala response to negative emotional stimuli after one night of sleep deprivation (Yoo et al., 2007). Similarly, Motomura and Mishima (2014) noted that poor sleep conditions increase vulnerability to negative emotions. It was found that emotional problems may affect one‘s quality and duration of sleep (Vandekerckhove et al., 2011). Negative mood states are associated with insomnia symptoms and sleep disturbance (Vargas et al., 2020), and ruminating over an experience of failure and bedtime worries about expected difficulties the next day negatively affected slow-wave sleep and its latency (Vandekerckhove et al., 2011). Thus, the relationship between sleep and emotion is bidirectional.

Obviously, sleep and emotion are important factors affecting false memory (Brainerd et al., 2008; Huan et al., 2021; Kersten et al., 2021; Newbury & Monaghan, 2019; Pardilla-Delgado & Payne, 2017). However, there is a lack of empirical research on the interaction effect of sleep and emotion on false memory. Most studies focused on the interaction effect of sleep and emotion on veridical memory. For example, Hu et al. (2006) investigated the consolidation of emotional episodic memory across 12 hours periods containing either a night of sleep or an equivalent period of time awake, and demonstrated that emotional memory is enhanced during sleep. Those who slept through the night remembered negative emotional images much better than those who had not slept during the day. But no such consolidation effect was found in the memory of neutral images. Gui and colleagues (2019) found that sleep helped consolidate positive emotional memories in healthy older adults, and this beneficial effect lasted for at least three days. Other studies also showed that sleep may preserve the emotional component of an experience during the consolidation of its content (e.g., Cellini et al., 2016; Werner et al., 2015). Such conservation of the emotional content after a sleeping period supports the hypothesis of the emotional salience view that the positive and negative memories are more susceptible to strengthening by sleep, as the specific neural network that is important for emotional processing and memory consolidation (the hippocampus-medial prefrontal cortex network) is more active during non-REM sleep (Genzel et al., 2015).

Evidence suggests that sleep and emotion interact to influence veridical memories, but precisely how these factors interact to affect false memory remains unresolved. Sleep is known to be critical for consolidating newly encoded situational events in both veridical memories (Weber et al., 2014) and false memories (Payne et al., 2009). However, McKeon et al. (2012) found that sleep would seem to consolidate these two types of memories in different ways when combined with the modulation of emotion. The study by McKeon et al. (2012) was to our knowledge the only work that focused on investigating the mechanism by which sleep–emotion affects false memory. They found increased false recall after sleep (compared to a waking group), regardless of whether the DRM word lists were emotionally negative or neutral. They argued that sleep did not simply reinforce memories, but rather supported their abstraction from the concrete learning context. However, McKeon et al. (2012) did not show significant differences between negative and neutral false memory after sleep. These results can be attributed to the use of emotional words in the study and their ability to trigger participants’ emotions. Therefore, more studies are needed to investigate the impact of sleep and emotion on false memory when precisely eliciting the emotional state of the participants, not just presenting emotional content.

To address the question of how sleep and varying the emotional context influence false memories, this study employs the DRM paradigm. For each experiment, we grouped words in the word lists by theme such that all words in each list were associated with a non-presented critical lure. Additionally, we used emotional words to elicit emotion during learning in Experiment 1 and video clips to induce a particular mood state before learning in Experiment 2. Previous studies had shown that negative emotions were difficult to manage, leading to the depletion of psychological resources (e.g., Vandekerckhove et al., 2011) and the increase of their cognitive load (e.g., Nabi, 1999). It was argued that sleep could consolidate the verbatim trace of the studied words including sensory details, therefore reducing false memory (Fenn et al., 2009). Taken together, negative emotion itself would occupy psychological resources, and thus would attenuate the effect of sleep on false memory. Therefore, we predicted that sleep and emotion would interactively affect false memory when participants are presented with negative words in the learning session (Experiment

1) or when their emotional state was induced before the learning session (Experiment 2).

## Experiment 1

### Participants

Twenty-four college students (10 males; mean age = 19.38 ± 1.44 years) were recruited and randomly assigned to the wake group or the sleep group. Potential participants were screened using an online survey to assess eligibility. After initial eligibility was established, the participants came to the psychology laboratory at South China Normal University to complete the sleep questionnaires and a DRM task. All participants had normal or corrected-to-normal vision, with no history of sleep disorder, neurological disease or head injury, and were not taking any medications affecting sleep. They provided informed written consent and received payment for participation.

### Materials

#### Sleep Questionnaires

Two scales were used to access the sleep characteristics of participants, the Morningness-Eveningness Questionnaire (MEQ; Horne & Ostberg, 1976) and the Pittsburgh Sleep Quality Index (PSQI; Buysse et al., 1989).

The MEQ consisted of 19 items and assessed chronotype. Most questions in the MEQ are designed in a preferential manner, whereby respondents are asked to indicate their preferred time of rising and bedtime, as well as physical and mental performance and alertness after rising and after different activities. This questionnaire includes five behavioral types: definitive morning (score = 70–86), moderate morning (score = 59–69), neither type (score = 42–58), moderate evening (score = 31–41), and definitive evening (score = 16–30).

The PSQI was used to measure subjective sleep quality. The measure includes 19 items related to the experience of sleep quality during the past 30 days. In all, 15 questions were rated on a four-point (0–3) Likert scale. The remaining four questions had open-ended response alternatives, which were rated on the same four-point (0–3) Likert scale based on the options. A global composite score was calculated based on the participant’s answers ranging from 0 (good sleep quality) to 21 (poor sleep quality).

#### DRM Task

The paradigm was based on the DRM word recognition task used by Roediger and McDermott (1995) to induce false memory. We selected 14 DRM word lists of 12 words from Cheng (2006), Huang (2010), Roediger and McDermott (1995), Stadler et al. (1999), and Zhang et al. (2012), which include seven negative word lists and seven neutral word lists. In each list, 11 words were to be the studied words and one was a critical lure (a word that semantically connects the words in each list). Ten lists served as learning materials in the learning phase, and the remaining four lists served as new items in the test phase. The learning phase contained five negative DRM word lists and five neutral DRM word lists except the critical lures (55 words in total). The test phase included 20 studied words, which were selected from the learning phase with two words from each DRM word list, 10 critical lures, and 20 unstudied words (serving as new items) from the extra four DRM word lists which did not appear in the learning phase, with five words from each word list. All the words were two-character Chinese words.

Sixteen extra subjects were asked to evaluate all the words according to the following four criteria: (a) emotional valence status: neutral or negative words; (b) emotional arousal: very low to very high arousal (1–9 scale); (c) familiarity: least to most familiar (1–9 scale); and (d) backward association strength (BAS; the subjects were asked to rate the tendency of associating the present word with the critical lure in each DRM word list): very low BAS to very high BAS (1–9 scale).

The proportion of negative studied words that were rated as negative valence was 92.62%, the negative critical lures were 100%, and the negative unstudied words were 91.83%. Additionally, the proportion of neutral studied words rated as neutral valence was 95.45%, the neutral critical lures were 98.75%, and the neutral unstudied words were 88.70%. Such results confirm the manipulation of negative and neutral words in the current study.

The arousal analysis showed that the arousal of negative words (5.09) was significantly higher than neutral words (2.95; F(1, 15) = 23.98, p < .001). However, there was no significant difference in arousal among negative studied words, negative critical lure, and negative unstudied words (F(2, 30) = 1.30, p = .29), nor among neutral studied words, neutral critical lure, and neutral unstudied words (F(2, 30) = .41, p = .67). The familiarity analysis showed that there was no significant difference between negative words (7.01) and neutral words (7.09; F(1, 15)= .03, p = .56). However, there was a significant difference among the familiarity of critical lures (7.61), studied words (7.03) and unstudied words (7.01). Fisher’s Least Significant Difference (LSD) post-hoc test showed higher familiarity for critical lures than for studied words (p = .003) and unstudied words (p = .02), with studied words and unstudied words being comparable (p = .94). The BAS analysis showed that there was no significant difference between negative (7.50) and neutral (7.66) words, F(1, 15) = .95, p = .35.

### Procedure

**Figure 1.**
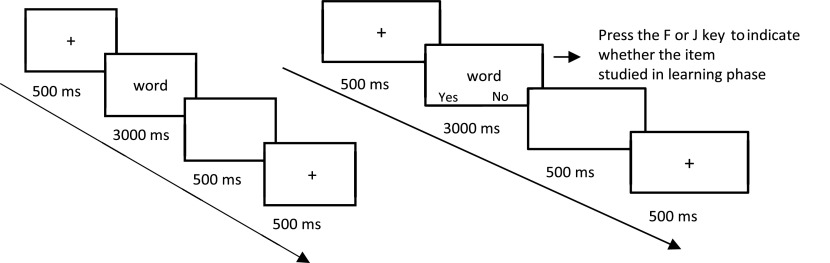
(a) Trial schematic for the word learning session; (b) Trial schematic for the recognition task

Before the start of the DRM experiment, participants signed the consent form and answered the sleep questionnaires. They then proceeded with the next step to complete the DRM experiment, which included a learning phase and a test phase. In the learning phase, participants completed the word learning task. Trials in the word learning task were completed in 10 blocks, with each block involving 11 words from one DRM word list. That is, participants studied negative words in five negative blocks (items in each block were from one of five negative DRM word lists) and neutral words in five neutral blocks (items in each block were from one of five neutral DRM word lists). Each trial started with a central cross displayed on the screen for 500 ms. After the presentation of the fixation point, each word was shown onscreen for 3,000 ms followed by a blank screen for 500 ms (see Figure 1a). In order to avoid the potential interference of emotion on the learning of neutral words, participants learned neutral word lists first and then learned negative word lists. Participants learned all 10 lists of words and then completed the recognition task 12 hours later (see Figure 1b). Words were presented to participants one at a time. Participants responded “yes” or “no” with a key press, to indicate whether each item had been presented by the computer in the learning phase.

Participants in the wake group completed the word learning task at around 9:00 a.m. and the recognition task at 9:00 p.m. on the same day. They were asked to avoid daytime napping during the retention interval. Participants in the sleep group completed the word learning task at around 9:00 p.m. and the recognition task at 9:00 a.m. on the next day. They were asked to get enough sleep during the night.

### Results of Experiment 1

#### Sleep Characteristics

There were no significant difference in PSQI scores (F(1, 22) = 1.57, p = .22) between wake and sleep groups, nor in MEQ scores (F(1, 22) = .02, p = .88).

#### Recognition Performance

The recognition rate of studied words was the proportion of correctly judged studied words; the false alarm rate of critical lures referred to the proportion of lures judged as studied words; and the false alarm rate of unstudied words referred to the proportion of unstudied words judged as studied words. False alarm rates of critical lures and unstudied words were misidentification rates. Table 1 shows the recognition rates and false alarm rates in the wake and sleep groups.

**Table 1:** Mean Recognition Rates of Studied Words and False Alarm Rates of Critical Lures and Unstudied Words in Experiment 1 (M ± SD)

		studied words	critical lures	unstudied words
Wake Group	negative	.75 ± .17	.85 ± .17	.23 ± .27
(N = 12)	neutral	.70 ± .18	.65 ± .12	.07 ± .13
Sleep Group	negative	.65 ± .23	.68 ± .16	.13 ± .18
(N = 12)	neutral	.63 ± .18	.68 ± .16	.03 ± .08

The rates at which words were recognized as studied words (i.e., recognition rates of studied words and false alarm rates of critical lures and unstudied words) were submitted to a 2 (Group: wake vs. sleep) × 2 (Word Affect Type: negative vs. neutral) × 3 (Word Type: studied words vs. critical lures vs. unstudied words) repeated-measures ANOVA, with Group as a between-subjects factor and Word Affect Type and Word Type as within-subjects factors. There was a significant main effect of Word Affect Type (*F*(1, 22) = 9.80, *p* = .01, *η^2^_p_* = .31), with the rate of recognition as studied words of negative words (.55) being higher than that of neutral words (.46). The main effect of Word Type was also significant (*F*(2, 44) = 169.65, *p* < .001, *η^2^_p_* = .89). Fisher’s Least Significant Difference (LSD) post-hoc test showed that the recognition rate of studied words (.68) and false alarm rate of critical lures (.72) were higher than the false alarm rate of unstudied words (.12; *ps* < .001), respectively, while there was no significant difference between critical lures and studied words (*p* = .35). However, the main effect of Group (*F*(1, 22) = 3.8, *p* = .06, *η^2^_p_* = .15), Group × Word Type interaction (*F*(2, 44) = .04, *p* = .97, *η^2^_p_* = .002), Group × Word Affect Type interaction (*F*(1, 22) = 3.10, *p* = .09, *η^2^_p_* = .12), Word Type × Word Affect Type interaction (*F*(2, 44) = 1.49, *p* = .24, *η^2^_p_* = .06), and Group × Word Type × Word Affect Type interaction (*F*(2, 44) = 1.12, *p* = .34, *η^2^_p_* = .05) were not significant.

To further explore the effect of Group and Emotional Valence, a 2 (Group: Wake vs. Sleep) × 2 (Word Affect Type: negative vs. neutral) repeated measures ANOVA was performed to analyze the recognition rate of studied words, false alarm rate of critical lures and unstudied words, with Group as a between-subject factor and Word Affect Type as a within-subject factor. For studied words, the main effects of Word Affect Type and Group, and the interaction effect between Group and Word Affect Type were not significant (*F*(1, 22) = .63, *p* = .44, *η^2^_p_* = .03; *F*(1, 22) = 1.60, *p* = .22, *η^2^_p_* = .07; *F*(1, 22) = .16, *p* = .70 , *η^2^_p_* = .01) respectively.

For critical lures, the main effect of Word Affect Type was significant (*F*(1, 22) = 5.50, *p* = .03 , *η^2^_p_* = .20) with a significantly higher false alarm rate for negative critical lures (.77) compared with neutral critical lures (.67). However, the main effect of Group was not significant (*F*(1, 22) = 2.05, *p* = .17, *η^2^_p_* = .09). Importantly, there was a significant interaction effect of Group and Word Affect Type (*F*(1, 22) = 5.50, *p* = .03 , *η^2^_p_* = .20). The false alarm rate of the wake group (.65) was comparable to the sleep group (.68) for neutral critical lures, *F*(1, 22) = .33, *p* = .57 , *η^2^_p_* = .02, but significantly higher than the sleep group for negative critical lures (.85 vs. .68), *F*(1, 22) = 6.04, *p* = .02, *η^2^_p_* = .22. Additionally, the false alarm rate of negative critical lures (.85) was higher than that for neutral ones (.65) in wake group, *F*(1, 11) = 9.43, *p* = .01, *η^2^_p_* = .46, while no such difference was found in the sleep group (.68 vs. .68).

**Figure 2.**
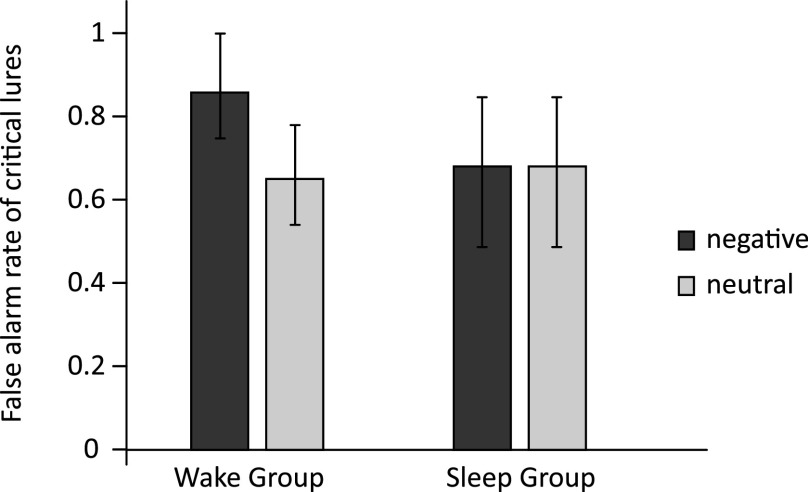
False alarm rate of critical lures for the Wake and Sleep Groups. Error bars represent standard error of the mean value.

For unstudied words, the main effect of Word Affect Type was significant, *F*(1, 22) = 7.65, *p* = .01, *η^2^_p_* = .26, with a higher rate for the false alarm of negative unstudied words (.18) than that for neutral unstudied words (.05). However, the main effect of Group and the interaction of Group and Emotional Type were non-significant, respectively (*F*(1, 22) = 1.52, *p* = .23, *η^2^_p_* = .07; *F*(1, 22) = .48, *p* = .50, *η^2^_p_* = .02).

### Discussion

Consistent with previous studies (e.g., Gallo & Roediger, 2002), our results showed that participants produced more false memories for critical lures than for unstudied words, indicating that the manipulation of false memory was successful. Compared with the sleep group, the wake group showed more false memories of the negative lures than for the neutral lures. That is, sleep and emotion have important impacts on false memory. The interpretation of these results will be discussed in the general discussion section. It should be noted that like the overwhelming majority of previous studies, Experiment 1 linked valence state with emotions using words. That is, Experiment 1 assessed selectively the emotional impact elicited by the affective words on false memory. Thus, it is difficult to differentiate whether the observed emotional effect stems from the affective word itself, or from the emotional states of participants when encoding the words. Experiment 2 reexamined the roles of sleep and emotion in false memory when a participant’s emotional state was directly elicited in the study phase.

## Experiment 2

### Participants

Eighty-eight college students were recruited and were randomly assigned to six groups (crossing two sleep conditions with three mood states). All participants had normal or corrected-to-normal vision with no history of sleep disorder, neurological disease or head injury. They were not taking sleep-affecting medications. Sixteen students were excluded for failing to complete the whole experiment. Finally, 12 participants participated in the sleep-positive condition, 11 in the sleep-negative condition, 12 in the sleep-neutral condition, 13 in the wake-positive condition, 12 in the wake-negative condition, and 12 in the wake-neutral condition. Thus, data analysis was performed on 72 participants (31 males; mean age = 19.78 ± 1.33 years).

### Materials

#### Sleep Questionnaires

Sleep quality was measured by the same scales as in Experiment 1.

#### DRM Task

Fourteen neutral word lists were used in Experiment 2. Each list was composed of 12 semantically related words, among which 11 words were used to be studied words and one as a critical lure (a word that semantically connects the words in each list). All the words were two-character Chinese words.

The same extra 16 subjects from Experiment 1 were asked to evaluate all the words for their emotional valence status, emotional arousal, familiarity, and BAS. The proportion of neutral studied words, neutral critical lures, and unstudied words being rated as neutral valence were 92.78%, 94.38%, and 94.11%. The arousal analysis showed that there was no significant difference in arousal among studied words (2.52), critical lures (2.56), and unstudied words (2.87), F(2, 30) = 3.18, p = .06. The familiarity analysis showed that there was significant difference among the critical lure, studied words, and unstudied words (F(2, 30) = 14.68, p < .001). Post-hoc tests showed greater familiarity for critical lures (7.72) than studied words (6.89, p < .001) and unstudied words (6.78; p = .001), with studied words and unstudied words being comparable (p = .56). The BAS analysis showed that there was no significant difference between studied words (7.72) and unstudied words (7.58; F(1, 15) = 1.23, p = .29).

#### Mood Induction Videos

Three videos were created to induce negative, positive and neutral states: a tragedy clip, a comedy clip, and a landscape clip. Twenty-seven additional college students were recruited to rate the emotional valence and arousal of the videos (nine participants for each video). Participants rated the emotional valence of the videos by judging whether it is negative, neutral or positive, and rated emotional arousal based on a nine-point Likert scale (1 for very low and 9 for very high).

For emotional valence, the negative video was judged as “negative” with the proportion of 100%, the positive video as “positive” with 100%, and the neutral video as “neutral” with 100%. The results indicated that the emotional valence among these videos was distinguishable.

A one-way ANOVA was performed to analyze emotional arousal. The results revealed that there was a significant difference among three videos in emotional arousal, F(2, 24) = 23.02, p < .001. Specifically, a post-hoc test showed that emotional arousal of the neutral video (4.67) was lower than the negative video (7.44; p < .001) and the positive video (7.67; p < .001), but there was no difference between the negative and the positive video (7.38 vs. 7.67; p = .58).

#### Mood Measure

Ten emotional words with five positives (interested, excited, enthusiastic, proud, active) and five negatives (distressed, scared, hostile, irritable, jittery) were adopted from the Positive and Negative Affect Scale (PANAS; Watson et al., 1988) to measure the participants’ mood states. For each item (e.g., happy) participants rated to what extent they felt this way at this moment on a five-point scale (from very slightly or not at all to extremely). A higher score indicated more of a corresponding affect.

### Procedure

The procedure was the same as in Experiment 1, with the exception of mood induction and measurement. Before the learning session, participants were instructed to watch a video (one third of the wake group and of the sleep group watched the positive video, another one third of the wake group and of the sleep group watched the neutral video, and the rest of the wake group and the sleep group watched the negative video). After watching the video, participants began the learning session and finished the PANAS scale as soon as they had completed learning the words. The test session was the same as in Experiment 1.

### Results of Experiment 2

#### Sleep Characteristics and Mood Manipulation Check

PSQI scores (F (5, 66) = 1.00, p = .14) and MEQ scores (F (5, 66) = 1.16, p = .52) did not differ across the six groups. Thus, the six groups showed similar sleep characteristics.

For the positive mood state, there was a significant difference among three mood state groups (F(2, 69) = 25.83, p < .001). A post-hoc test showed that participants who watched the positive video (positive group; 15.92) had higher ratings of positive mood state than those who had watched the neutral video (neutral group; 12.58; p < .001) and those who watched the negative video (negative group; 9.50; p < .001. And the neutral group got higher scores than the negative group (p = .001).

For the negative mood state, there was a significant difference among the three mood state groups (F (2, 69) = 54.49, p < .001). A post-hoc test showed that participants in the negative group (15.00) had higher ratings of negative mood state than those of the neutral group (6.29; p < .001) and those of the positive group (5.96; p < .001). But there was no significant difference between the negative group and the neutral group, p = .73. The results indicated that the participants’ emotional states were successfully manipulated.

#### Recognition Performance

*Table 2* shows the recognition rate of the three types of words in each group.

**Table 2:** Mean Recognition Rates of Studied Words and False Alarm Rates of Critical Lures and Unstudied Words in Experiment 2 (M ± SD)

		studied words	critical lures	unstudied words
Wake Group	negative (N = mood 12)	.74 ± .12	.85 ± .10	.22 ± .20
	positive (N = mood 13)	.69 ± .18	.77 ± .11	.20 ± .17
	neutral (N = mood 12)	.68 ± .19	.78 ± .16	.18 ± .13
Sleep Group	negative (N = mood 11)	.69 ± .16	.71 ± .17	.16 ± .14
	positive (N = mood 12)	.81 ± .20	.61 ± .23	.11 ±.10
	neutral (N = mood 12)	.73 ± .09	.81 ± .16	.21 ± .14

A 2 (Group: Wake vs. Sleep) × 3 (Mood State: positive vs. neutral vs. negative) × 3 (Word Type: studied words vs. critical lures vs. unstudied words) ANOVA was conducted to analyze recognition rate, with Group and Mood State as between-subject factors and Word Type as a within-subject factor. The results revealed that neither the main effect of Group (*F*(1, 66) = 1.266, *p* = .27, *η_^2^_p* = .02), nor the main effect of Mood State (*F*(2, 66) = .60, *p* = .55, *η^2^_p_* = .02) was significant.

The main effect of Word Type was significant (*F*(2, 132) = 496.85, *p* < .001, *η^2^_p_* = .88). Specifically, the post-hoc test showed that the false alarm rate of critical lures (.75) and the recognition rate of studied words (.72) were higher than the false alarm rate of unstudied words (.18) (*ps* < .001), while there was no significant difference between critical lures and studied words (*p* = .13).

The Group × Mood interaction effect was not significant (*F*(2, 66) = 1.36, *p* = .26, *η^2^_p_* = .04). However, the Group × Word Type interaction effect was significant (*F*(2, 132) = 5.17, *p* = .01, *η^2^_p_* = .07). There was no significant difference between the wake group and the sleep group for the studied words (.70 vs. .74; *F*(1, 70) = 1.12, *p* = .29, *η^2^_p_* = .02), nor for unstudied words (.20 vs. .16; *F*(1, 70) = 1.45, *p* = .23, *η^2^_p_* = .02). For critical lures, the wake group had a higher false alarm rate (.80) than the sleep group (.71; *F*(1, 70) = 5.28, *p* = .03 , *η^2^_p_* = .07).

Further, the interaction between Word Type and Mood State was significant (*F*(4, 132) = 2.83, *p* = .03, *η^2^_p_* = .08). There was no significant difference among neutral mood condition (.71), positive mood condition (.75), and negative mood condition for the studied words (.71; *F*(2, 69) = .51, *p* = .60, *η^2^_p_* = .02), nor for unstudied words (.20 vs. .15 vs. .19; *F*(2, 69) = .59, *p* = .56, *η^2^_p_* = .02). For critical lures, the main effect of Mood State was marginally significant (*F*(2, 69) = 2.76, *p* = .07, *η^2^_p_* = .07). Post-hoc tests showed that the false alarm rate in the positive state condition (.69) was lower than that in the neutral state condition (.80; *p* = .03). No other difference was found between the positive and negative state conditions (.69 vs. .78; *p* = .07), nor between the neutral and negative state conditions (.80 vs. .78; *p* = .76).

More importantly, the Group × Mood State × Word Type interaction was marginally significant, *F*(4, 132) = 2.18, *p* = .08, *η^2^_p_* = .06. For the studied words, the main effects of Mood State and Group, and the interaction effect between Mood State and Group were not significant (*F*(2, 66) = .59, *p* = .56, *η^2^_p_* = .02; *F*(1,66) = 1.02, *p* = .32, *η^2^_p_* = .02; *F*(2,66) = 1.6, *p* = .22 , *η^2^_p_* = .05). Similar results were found for unstudied words (*F*(2, 66) = .63, *p* = .54, *η^2^_p_* = .02; *F*(1, 66) = 1.44, *p* = .24, *η^2^_p_* = .02; *F*(2, 66) = .89, *p* = .41, *η^2^_p_* = .03). For critical lures, the false alarm rate in the wake group was higher than that of the sleep group in the positive state condition (.77 vs. .61; *F*(1, 23) = 5.11, *p* = .03, *η^2^_p_* = .18) and the negative state condition (.85 vs. .71; *F*(1, 21) = 5.92, *p* = .02, *η^2^_p_* = .22). However, there was no significant difference between the sleep group (.81) and the wake group (.78; *F*(1, 22) = .17, *p* = .68, *η^2^_p_* = .01) in the neutral state condition.

**Figure 3.**
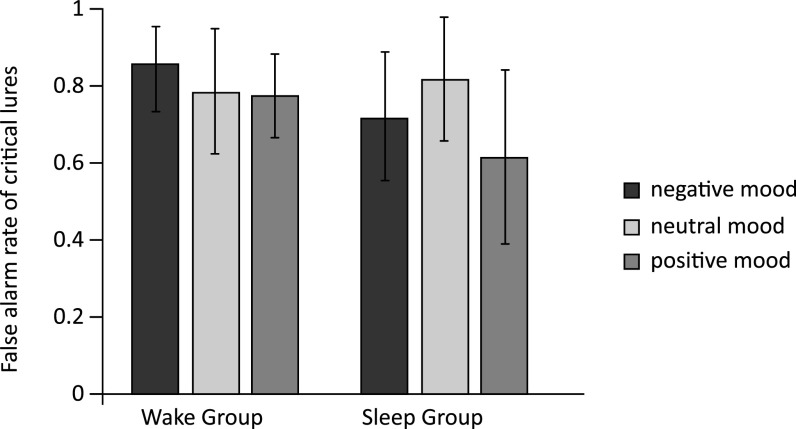
False alarm rate of critical lures in Experiment 2. Error bars represent standard error of the mean value.

### Discussion of Experiment 2

Similar to Experiment 1, the false alarm rate was higher for the critical lures compared to the unstudied words in all groups, suggesting successful induction of false memory in the current experiment, which is consistent with previous research (e.g., Gallo & Roediger, 2002). There was a significant difference in the false alarm rate of critical lures between the sleep and wake groups in the positive and negative mood state conditions, indicating that sleep and mood state would impact false memory. These will be discussed in more detail below.

## General Discussion

Given the discrepancies in earlier findings on the effects of emotion and sleep on memory formation (Ashton et al., 2020; Bookbinder, & Brainerd, 2017; Pourtois et al., 2013), the present study addressed the question whether sleep and emotion would have an interactive effect on the formation of false memory, using emotional words and mood state induction. The results of Experiment 1 showed that the wake group produced more false memory (a higher false alarm rate of critical lures) than the sleep group when words expressed a negative meaning, but no such significant difference between the wake and sleep groups for neutral words. This increase of false memory in the wake group was again present when participants were experiencing a positive or negative emotional state in Experiment 2.

Our finding that participants in the wake group showed significantly greater false memory of negative words is in line with previous studies (e.g., Brainerd et al., 2008; Sharkawy et al., 2008). When participants were presented with negative words, information about valence was evoked (Gianotti et al., 2008). The false memory of negative words or when participants were experiencing negative emotion is greater than that of neutral words, which may be because of the influence of emotion delivery (i.e., negative emotion was stronger than neutral emotion). This seemingly supported fuzzy-trace theory, which suggested that gist retrieval may trigger false memory vulnerability in items that shared meanings. The emotional valence would strengthen the connection among words, which in turn enhanced gist trace of memory and thus led to more false memory (Howe et al., 2010; Zhang et al., 2017).

However, such a difference was only exhibited in the wake group, but not in the sleep group in the current study, which seems inconsistent with some previous studies. Previous evidence showed that false memory of critical lures increased after a period of sleep compared with being awake (e.g., Calvillo et al., 2016; Diekelmann et al., 2010; McKeon et al., 2012), or relative to being deprived of sleep (e.g., Chatburn et al., 2017). Those researchers argued that sleep could facilitate the generalization of related information, and thus gist memory could be more easily extracted. However, there were other studies showing no effects or even a reduction of the generation of gist memories after sleep compared to wakefulness (e.g., Fenn et al., 2009; Lo, Sim, & Chee, 2014). It was claimed that these mixed results using the DRM paradigm were partly due to differences between recognition versus recall tests, but also possibly due to the particular semantic properties of the DRM lists used (Monaghan et al., 2017). Thus, the effect of sleep on susceptibility to false memories was still unclear.

The findings of the current study suggest that the effect exerted by sleep on false memory was moderated by emotion. That is, the mechanism to account for observations of decreased false memories in the sleep group could be related to the emotional words that we chose or to our emotion induction design. When the words expressed a negative emotion, the subjects more likely retrieved the previous memory trace and encoded the words expressing the same negative affect type together, or even spread activation in semantic memory. On the one hand, evidence suggests that sleep could affect participants’ ability to generalize their knowledge of multiple items (Darsaud et al., 2011); on the other, it was found that sleep could consolidate item-specific details associated with veridical information, thereby enhancing source monitoring processes (Fenn et al., 2009). Following this line, sleep would decrease participants’ ability to spread the semantic knowledge of negative items by consolidating these learned items. It is also possible that sleep between exposure to DRM lists and testing could decrease the acceptance of negative lure words more than staying awake between sessions.

It is also possible that daytime wakefulness, while assisting episodic memory consolidation, might promote semantic processing. For recognition of both list and gist words, participants who remained awake during the daytime after encoding, compared to night sleep, relied on a broader, more distributed cortical network, suggesting that daytime wakefulness shifts the brain to more effortful strategies to retrieve information. Similarly, Diekelmann et al. (2008) deprived some participants of sleep before testing their memory and found an increase in false recognition of critical lures. We suspect that remaining wakeful (even in the daytime) helps extract the overall idea, or “gist,” of a task, from which the brain can identify common features of new waking experiences and incorporate them into a new schema.

Researchers have shown that different negative emotions (e.g., sadness, anger, fear) have different effects on cognitive processes such as attention and memory processing (e.g., Kaplan et al., 2016; Van Damme et al., 2017). Emotion has two distinct dimensions, arousal and valence, both of which influence memory (Brainerd et al., 2010). According to fuzzy-trace theory, the valence of critical distractors should have different effects on false memory, relying on the relation between critical lures and list words. For semantic lists, list words and critical lures are both valenced, so that emotional valence facilitates the extraction of the meaning relationship across list words (e.g., Bookbinder & Brainerd, 2017) by deflecting processing from the surface details of item presentations (e.g., Bookbinder & Brainerd, 2017).

Studies have also found that high arousal emotion states or learning materials can elicit more false memory regardless of their valence features (Corson & Verrier, 2007). This may be due to the high arousal content or states during encoding, which narrow the scope of attention and lead to concentrating on gist trace and neglecting peripheral details (e.g., Bookbinder & Brainerd, 2017; Kaplan et al., 2016). However, Bayer et al. (2010) showed that the Late Positive Component (LPC) was unaffected by arousal when valence was controlled. Though the available results seem mixed, most studies indicate the possibility that the ambiguity of emotional content (valence), as well as its intensity (arousal), determine how memory is influenced. As the arousal levels between negative and neutral words or across all three mood-induction groups were not well controlled in the present study, the mood effect that we found may have been due to valence-arousal effects rather than to a valence effect per se.

Emotion regulation theories of sleep argue that sleep is an important mechanism impacting emotional responses to stressors (Deliens et al., 2014; Goldstein & Walker, 2014). Motomura and Mishima (2014) suggested that poor sleep conditions could increase the vulnerability of negative emotions. Similarly, Baglioni et al. (2010) found that the sleep deprivation group displayed enhanced activity in the amygdala and reduced functional connectivity between the amygdala and the medial prefrontal cortex (PFC). That is, there was an increased neurobiological response to emotional stimuli and a reduced inhibitory influence of the PFC on emotional reactivity after sleep deprivation. Other neuroimaging studies also indicated that following a night of sleep deprivation, individuals showed an exaggerated amygdala response to negative emotional stimuli (e.g., Yoo et al., 2007). Other studies also showed that sleep is an important and modifiable factor that can influence emotion regulation (Tamanna et al., 2014). In the reverse direction, it is possible that emotion regulation can impact sleep. For example, poor emotion regulation can lead to mental health issues, which could worsen sleep quality (Gross & John, 2003). Based on previous literature showing that sleep and emotion affect each other mutually, the current study further extends these findings in that such sleep–emotion interaction could have long-term effects on false memory. Future work will be needed to confirm the current finding and further determine the extent that these two different factors might contribute to this process.

## Conclusion

This study provides further support for findings that sleep is an important mechanism impacting emotional responses (e.g., Goldstein & Walker, 2014). The main and novel finding of the study is that such sleep–emotion interaction affects false memory. The strength of the effect is reflected in the intended negative emotion elicited by the negative words during learning and the mood state (negative or positive) induced by video clips before learning. To our knowledge, this study is the first to reveal the roles of sleep and emotion in the formation of false memories by manipulating emotion during learning and before learning.

## Limitations

Two main limitations of this study are the marginal significance for the three-term interaction and the use of a statistical significance level of *p* < 0.05 (uncorrected). We used the LSD post-hoc test to slightly control type I error. For full consideration of multiple comparisons, more stringent correction such as Bonferroni or false discovery rate correction should be applied. As can be seen from the results, only two of the three “significant” results in Experiment 1 and three of the six “significant” results in Experiment 2 based on the critical multiple comparisons survived this more stringent criterion. Further studies are required to address these considerations by increasing statistical test power with a large sample size.

## Appendix 1. DRM Word Lists Used in Experiment 1

**Table A1:** Negative DRM Lists Used in Experiment 1

DRM Lists	1		2		3		4		5	
critical lures	生气	anger	犯罪	crime	烦闷	worried	仇恨	hostility	畏惧	anger
studied words	狂怒	mad	罪犯	criminal	孤独	lonely	讨厌	hate	胆怯	mad
	害怕	fear	罪恶	evil	哀愁	sad	厌恶	abhorrent	害怕	fear
	憎恨	hate	盗窃	steal	苦闷	agonizing	痛恨	abomination	畏怯	hate
	大怒	rage	骚扰	harass	郁闷	depressed	虚伪	hypocrisy	怯懦	rage
	脾气	temper	绑架	kidnap	恼火	irritated	报复	revenge	恐惧	temper
	暴怒	fury	破坏	destroy	沮丧	dispirited	憎恨	detest	胆小	fury
	忿怒	ire	小偷	thief	失意	frustrated	嫉妒	envy	胆寒	ire
	愤怒	wrath	抢劫	rob	自卑	self- abasement	怨恨	resentment	畏忌	wrath
	争论	fight	凶手	murderer	烦恼	annoyed	战争	war	忌惮	fight
	激怒	enrage	谋杀	murder	抱怨	complaining	愤恨	resentful	后怕	enrage
	暴躁	fiery	拐卖	abduction	烦躁	whiny	冤仇	enmity	畏缩	fiery

**Table A2:** Neutral DRM Lists Used in Experiment 1

DRM Lists	6		7		8		9		10	
critical lures	音乐	music	双脚	foot	椅子	chair	女孩	girl	国王	king
studied words	音符	note	鞋子	shoe	桌子	table	芭比	dolls	王后	queen
	声音	sound	双手	hand	桌腿	legs	女性	female	王冠	crown
	钢琴	piano	脚趾	toe	座位	seat	年轻	young	王子	prince
	唱歌	sing	凉鞋	sandals	卧榻	couch	女装	dress	女王	empress
	广播	radio	足球	soccer	睡椅	recliner	漂亮	pretty	权利	right
	乐队	band	鞋码	yard	沙发	sofa	头发	hair	宫殿	palace
	旋律	melody	走路	walk	木头	wood	侄女	niece	王位	throne
	乐器	instru- ment	脚踝	ankle	坐垫	cushion	舞蹈	dance	统治	rule
	和声	harmony	靴子	boot	凳子	stool	可爱	cute	臣民	subjects
	爵士	jazz	短袜	sock	摇椅	rocking	阿姨	aunt	君王	monarch
	韵律	rhythm	趾甲	nail	长椅	bench	姐妹	sister	王室	royal

**Table A3:** Negative and Neutral Unstudied DRM Lists Used in Experiment 1

DRM Lists	11		12 words		13		14 words	
	negative unstudied				neutral unstudied			
	苦闷	agonizing	愚蠢	silly	眼睛	eye	文具	stationery
	忧郁	melancholy	笨拙	stupid	眼镜	glasses	钢笔	pen
	愧疚	guilty	愚昧	ignorance	视觉	vision	学习	learning
	苦恼	agonizing	愚钝	crass	近视	myopia	墨水	ink
	抑郁	despondent	愚笨	foolish	镜片	lens	铅笔	pencil
	忧愁	woebegone	蠢笨	clumsy	视线	sight	橡皮	eraser
	愧恨	ashamed	拙笨	unskillful	眼球	eyeball	笔袋	bag
	愁苦	anxiety	智障	amentia	眼科	ophthalmology	书包	schoolbag
	忧闷	gloomy	迟钝	dull	眼神	look	直尺	ruler
	忧恼	sorrow	痴呆	oafish	视野	view	毛笔	brush
	歉疚	sorry	呆板	inflexible	光学	opics	胶带	tape
	苦痛	pain	弱智	retarded	散光	astigmatism	书本	book

## Appendix 2. DRM Word Lists Used in Experiment 2

**Table A4:** Neutral DRM Lists Used in Experiment 2

DRM Lists	1		2		3		4		5	
critical lures	箱子	box	双脚	foot	国王	king	文具	stationery	眼睛	eye
studied words	包装	pack	鞋子	shoe	王后	queen	钢笔	pen	眼镜	glasses
	储藏	store	双手	hand	王冠	crown	学习	learning	视觉	vision
	盒子	case	脚趾	toe	王子	prince	墨水	ink	近视	myopia
	纸盒	carton	凉鞋	sandals	女王	empress	铅笔	pencil	镜片	lens
	工具	tool	足球	soccer	权利	right	橡皮	eraser	视线	sight
	衣箱	suitcase	鞋码	yard	宫殿	palace	笔袋	bag	眼球	eyeball
	方形	cube	走路	walk	王位	throne	书包	school- bag	眼科	ophthalmology
	皮箱	luggage	脚踝	ankle	统治	rule	直尺	ruler	眼神	look
	木匣	affair	靴子	boot	臣民	subjects	毛笔	brush	视野	view
	鞋盒	shoe- box	短袜	sock	君王	monarch	胶带	tape	光学	opics
	容器	container	趾甲	nail	王室	royal	书本	book	散光	astigmatism

**Table A5:** Neutral DRM Lists Used in Experiment 2

DRM Lists	6		7		8		9		10	
Critical lures	河流	river	窗户	window	衣服	clothing	化学	chemistry	房子	house
Studied words	流水	water	门窗	door	时装	fashion	烧杯	beaker	建筑	architecture
	溪流	stream	玻璃	glass	服装	clothes	元素	element	楼层	floor
	长江	Mississippi	窗台	sill	衬衣	shirt	物理	physics	楼房	building
	小船	boat	房间	house	西装	suit	实验	experiment	修建	build
	游泳	swim	窗帘	curtain	风衣	coat	溶液	solution	修筑	construct
	流动	run	窗框	frame	背心	vest	分子	molecule	阁楼	attic
	小溪	creek	视野	view	大衣	overcoat	烧瓶	flask	房舍	premises
	小河	brook	通风	breeze	帽子	cap	有机	organic	楼梯	stairs
	鱼类	fish	纱窗	screen	夹克	jacket	氧化	oxidation	装修	decoration
	桥梁	bridge	窗花	paper-cutting	毛衣	sweater	蒸馏	distill	房产	property
	蜿蜒	winding	橱窗	shop- window	裤子	pants	滴管	dropper	书房	study

**Table A6:** Neutral DRM Unstudied Lists Used in Experiment 2

DRM Lists	11		12		13		14	
unstudied words	山脉	mountain	时间	time	面包	bread	车辆	vehicle
	丘陵	hill	时刻	moment	黄油	butter	公路	highway
	山谷	valley	时光	days	早餐	breakfast	汽车	automobile
	登山	climb	瞬间	instant	黑麦	rye	卡车	truck
	山顶	summit	世纪	century	果酱	jam	驾驶	drive
	顶峰	top	日期	date	牛奶	milk	轮胎	tire
	山丘	molehill	一刻	quarter	面粉	flour	公车	bus
	山峰	peak	手表	watch	奶酪	cheese	赛车	race
	平原	plain	间隔	interval	面团	dough	货车	van
	雪山	glacier	岁月	years	吐司	toast	吉普	jeep
	陡峭	steep	毫秒	millisecond	奶油	cream	车程	range
	山岗	molehill	钟表	clock	甜点	dessert	车库	garage
